# Cross-Domain Generalization of Deep Learning Architectures for Cephalometric Landmark Detection: A Dual-Dataset and Multi-Device Benchmark

**DOI:** 10.3390/diagnostics16172726

**Published:** 2026-08-26

**Authors:** Mustafa Özcan, Ferdi Allaf

**Affiliations:** Department of Orthodontics, Faculty of Dentistry, Istanbul Health and Technology University, 34275 Istanbul, Türkiye; ferdi.allaf@istun.edu.tr

**Keywords:** cephalometry, anatomic landmarks, orthodontics, domain shift, cross-device generalization, external validation, benchmarking

## Abstract

**Background/Objectives:** Deep learning models for cephalometric landmark detection report near-ceiling accuracy on single benchmarks, yet most are trained and tested on the same dataset. Whether the best in-domain architecture remains best out-of-domain has not been systematically quantified. **Methods:** Four architecture families (heatmap CNN, two-stage cascade, coordinate-regression Vision Transformer, pretrained ResNet-50) were each trained on two independently sourced datasets—ISBI 2015 (400 images, one device) and Aariz (1000 images, seven devices)—and evaluated on both, over their 19 shared landmarks in millimeters. All axes used three seeds (mean ± SD): the cross-dataset matrix, leave-one-device-out shift, balanced joint training, a pretrained-versus-scratch ablation, and a landmark-level breakdown. **Results:** In-domain mean radial error (MRE) was 2.47–3.04 mm (Aariz) and 4.12–5.55 mm (ISBI); cross-dataset error rose steeply (Generalization Drop—the relative increase in error out-of-domain—155–426%). The most accurate model in-domain (a from-scratch heatmap CNN, 2.47 mm) showed the largest drop, and every pretrained estimate fell below every from-scratch estimate (family means 248% vs. 380%): in-domain ranking did not predict cross-domain ranking. Leave-one-device-out revealed reproducible device-specific shift (held-out MRE 1.89–7.94 mm). Inter-observer variability was 0.53 mm, so cross-domain errors were 15–44× the human band. Balanced joint training reduced the cross-domain gap for all four architectures (both domains ≈ 2.1–3.5 mm) without harming in-domain accuracy. Pretraining more than halved cross-domain error from Aariz to ISBI (7.73 vs. 16.36 mm) but not in reverse, supporting the mechanism in one direction rather than uniformly. A-point, B-point, Nasion, and Menton all exceeded the 2 mm clinical threshold out-of-domain, including points localized to sub-millimeter accuracy in-domain. **Conclusions:** Single-dataset accuracy substantially overstates clinical generalizability, and the best in-domain architecture is not the most transferable, so in-domain leaderboards are an unreliable basis for selecting a model for clinical deployment; balanced multi-source training recovers most of the loss across the architectures tested.

## 1. Introduction

Cephalometric analysis underpins orthodontic diagnosis and treatment planning, and the manual tracing of anatomical landmarks on lateral cephalograms is time-consuming and subject to inter- and intra-observer variability [1,2]. Automated landmark detection has, therefore, been pursued intensively, and on the canonical ISBI 2015 benchmark, recent methods report mean radial errors approaching expert-level precision [3,4].

Landmark localization accuracy directly propagates into every downstream cephalometric measurement: an error of a few millimeters at a single point can shift the angular measurements used for skeletal classification, such as ANB and SNA, by clinically meaningful amounts [1,2], altering skeletal classification and, in turn, treatment decisions; AI-driven cephalometric analysis is increasingly applied to such downstream tasks, including longitudinal growth assessment [5]. This sensitivity is why sub-millimeter and low-millimeter accuracy has become the de facto target for automated systems, and why the field has converged on the mean radial error (MRE) and the success detection rate (SDR) at fixed millimetric thresholds as its primary metrics, the 2 mm threshold having been established by the ISBI 2015 challenge [3] and used throughout subsequent work [6]. Reported performance on ISBI 2015, however, is now so high that headline numbers alone reveal little about whether a model would remain reliable outside the conditions under which it was trained.

A structural limitation runs through much of this literature: models are typically trained and evaluated on a single dataset, most often the ISBI 2015 Grand Challenge set (IEEE International Symposium on Biomedical Imaging), which was acquired entirely on one device at a fixed 0.1 mm/pixel resolution. A model that excels on its own test split may degrade substantially on images from other scanners, resolutions, or populations, and this degradation is precisely what determines clinical usability. In practice, these factors co-occur: datasets that differ in acquisition equipment typically also differ in the patient populations they were drawn from, so the shift observed between any two public datasets is a composite one. Recent dataset work argues that single-device training data limit generalizability [7], and a 2025 scoping review identifies dataset standardization, barriers to clinical integration, and cross-architecture performance variability as core unresolved problems [6]. In medical imaging more broadly, this degradation under acquisition shift across devices and sites is well documented and termed domain shift [8,9].

Crucially, domain shift interacts with model architecture in ways that are not captured by in-domain benchmarks. Different architecture families encode different inductive biases: heatmap-regression convolutional neural networks (CNNs) localize points through dense spatial likelihood maps, coordinate-regression networks predict positions directly, cascaded designs refine coarse estimates in stages, and transformer backbones aggregate global context through self-attention. There is no a priori reason to expect that the inductive bias best suited to fitting a single dataset is also the one most resilient to acquisition shift; indeed, the two objectives may be in tension, since a model flexible enough to reach very low in-domain error can also overfit device-specific texture and intensity characteristics that do not transfer. Whether this tension exists in practice for cephalometric landmarking, and if so, how large it is, has not been quantified.

What is missing is a multi-architecture, multi-dataset, clinically calibrated (millimeter-scale) generalization benchmark. Our central question is whether the architecture that performs best on one dataset also performs best on another, and how architecture families—heatmap CNN, two-stage cascade, coordinate-regression Vision Transformer, and a pretrained ResNet-50 backbone—diverge under cross-dataset conditions. The contribution is not a new state-of-the-art model but a systematic quantification, for cephalometry specifically, of how far single-dataset accuracy overstates cross-domain performance, summarized through an interpretable measure we report as the Generalization Drop (GD), a relative form of the generalization gap between in- and out-of-domain error widely studied in machine learning [8].

Concretely, we make five contributions. First, we train four representative architecture families under a single fixed protocol on each of two independently sourced datasets and evaluate every trained model on both, yielding a full cross-dataset matrix in which architecture is the only deliberately varied factor. Second, we complement this with a within-dataset leave-one-device-out analysis across seven imaging devices, testing whether the same degradation appears at the finer granularity of scanner-to-scanner shift, and we anchor all errors against human inter-observer variability measured on the same landmarks. Third, we test a simple, deployable mitigation—balanced multi-source training—and quantify how much of the cross-domain loss it recovers for each architecture. Fourth, we isolate the contribution of ImageNet pretraining in a controlled ablation in which a single architecture is trained from pretrained and from random initialization, so that the mechanism behind the observed transfer advantage is tested rather than assumed. Fifth, we resolve the aggregate error into its 19 constituent landmarks for two architectures chosen to bracket the effect, so that the clinical reading of the results is anchored to the specific anatomical points on which diagnosis depends. Together, these quantify the cross-dataset and cross-device generalization gap for cephalometry, show how it behaves, and demonstrate that it can be substantially reduced.

## 2. Materials and Methods

### 2.1. Datasets

Two independently sourced, publicly available, landmark-annotated datasets acquired on different equipment were used. ISBI 2015 [3] includes 400 lateral cephalograms, 19 landmarks, a single device, a 0.1 mm/pixel, and official splits of 150 train/150 test1/100 test2. Aariz [7] includes 1000 cephalograms, 29 landmarks across seven imaging devices, with per-image pixel calibration, and splits of 700/150/150. Ground truth was the senior + junior mean on Aariz and the senior tracing on ISBI (the public export contains senior only). Aariz is distributed under the Creative Commons Attribution (CC-BY) license, and ISBI 2015 is likewise publicly available for research use. No data are redistributed; only the results and code are released, and both datasets are cited.

Both datasets are publicly available and were used in accordance with their licenses; no new patient data were generated in this study. Their provenance, expert validation, ethical status, and licensing are detailed below.

**ISBI 2015 dataset.** Source: IEEE International Symposium on Biomedical Imaging (ISBI) 2015 Grand Challenge (Wang et al. [3]); the dataset is openly distributed by the challenge organizers and was accessed via its public repository (accessed July 2026). It comprises 400 lateral cephalograms from 400 subjects (ages 6–60), acquired on a single Soredex CRANEX Excel unit and distributed by the challenge organizers at 1935 × 2400 pixels with a uniform 0.1 mm/pixel calibration; this is the challenge distribution format rather than a per-unit acquisition setting, with each annotated with 19 landmarks by two experienced clinicians. The senior tracing served as ground truth here, which is consistent with prior work. Annotations were produced and cross-checked by expert clinicians as described by the challenge organizers. The data were released as fully anonymized, open-access human-subject data by the IEEE ISBI 2015 Grand Challenge, and secondary analysis of this de-identified public dataset did not require institutional review board approval. The dataset is distributed under a Creative Commons license (CC BY-SA 4.0); our use—analysis without redistribution of the images—complies with these terms.

**Aariz dataset.** Source: Khalid et al. [7], Scientific Data 2025;12:1336, and deposited on Figshare (DOI: 10.6084/m9.figshare.27986417; accessed July 2026). The 1000 lateral cephalograms were curated from a larger pool of 3500 radiographs acquired on seven different imaging devices, contributed by clinicians at Islamic International Dental College with the support of Riphah International University, Islamabad, Pakistan. Each radiograph carries 29 landmarks; this study used the 19 landmarks common to both datasets, which were clinician-verified for anatomical correspondence. Annotations were produced by a team of six orthodontists in two phases (junior labeling followed by senior review), with a full expert re-review of the dataset to correct annotation errors, as reported by the dataset authors. The dataset was fully anonymized, with all personally identifiable information removed in compliance with ethical guidelines and images used with patient consent as reported by the dataset creators; secondary analysis of this de-identified public dataset did not require additional institutional review board approval. It is publicly available under a Creative Commons Attribution (CC-BY) license, and our use complies with these terms.

### 2.2. Shared-Landmark Mapping

The two datasets share 19 landmarks: every ISBI 2015 landmark has an anatomical equivalent in Aariz, exceeding the pre-specified ≥ 12 threshold. The full correspondence is given in Table 1; three potentially ambiguous mappings—the upper and lower lip and Soft Tissue Pogonion—were explicitly confirmed by an orthodontist (M.Ö.). All errors are reported in millimeters using each dataset’s pixel calibration (Aariz: per-image device spacing; ISBI: 0.1 mm/pixel). The coordinate loss was computed in network-resolution pixel space during training; for evaluation only, predictions and ground truth were rescaled from network resolution back to original image pixels and the per-image millimeter calibration was then applied, so reported errors are in true millimeters regardless of network input size.

### 2.3. Architectures and Training

Four families were compared under an identical training budget for fairness: a High-Resolution Network (HRNet)-style high-resolution heatmap network (parallel multi-resolution branches) [10] with soft-argmax (differentiable spatial-to-numerical) decoding [11]; a two-stage deep-supervised cascade [12]; a coordinate-regression Vision Transformer (ViT; vit_small_patch16_224) [13]; and an ImageNet-pretrained ResNet-50 [14] with a coordinate-regression head. Heatmap regression and CNN-based detectors are established for this task [15,16]. Inputs were 512 × 512 for heatmap families and 224 × 224 for the coordinate-regression families (the native resolution of the pretrained backbones); within the cross-dataset matrix, all models shared the same optimizer, schedule, loss, augmentation, and epoch count (60 epochs, Adam, cosine learning-rate decay, smooth-L1 loss, light brightness augmentation; batch size 6 for the 512 × 512 heatmap families and 8 for the 224 × 224 coordinate-regression families, chosen to fit the 16 GB unified memory of an Apple M2 Pro with Metal Performance Shaders acceleration). The joint-training, ablation, and landmark-level runs used the same 60-epoch budget; the leave-one-device-out runs, which involve a single architecture and, therefore, no cross-architecture comparison, used 50 epochs, as recorded in the released code. Holding the recipe fixed is deliberate and central to the design: because our question concerns the relative behavior of architectures rather than the maximum attainable accuracy of any one of them, per-architecture hyperparameter optimization would confound architecture with tuning effort and make cross-family comparison uninterpretable. A single shared recipe, therefore, compares architectures under equal experimenter effort rather than at their individual optima. We note explicitly that this is not the same as isolating architecture as a causal factor: different families have different optimal hyperparameters, and a fixed recipe will disadvantage some more than others, so the comparison should be read as behavior under a common budget. Absolute accuracy is, therefore, not optimized for any single model; notably, the coordinate-regression families generalized best despite their lower input resolution. Each of the eight train/test cells was repeated with three random seeds (seeds 0–2); the results are reported as mean ± SD across seeds. All models were implemented in Python 3.14.5 with PyTorch 2.12.0, timm 1.0.27, NumPy 2.4.6 and SciPy 1.17.1.

### 2.4. Evaluation and Design Axes

Each model was trained on each dataset and evaluated on the held-out test split of both, forming a 2 × 2 in-/cross-domain matrix. Although the field reports both MRE and SDR, we designate mean radial error (MRE, mm) as the primary metric here, together with the Generalization Drop, GD = (MRE_cross − MRE_in)/MRE_in × 100, because a continuous error measure is better suited to variance estimation across seeds than a thresholded success rate. The success detection rate (SDR) at the 2.0 mm clinically accepted precision range established by the ISBI 2015 challenge [3] (computed alongside 2.5/3.0/4.0 mm) is reported as a secondary, descriptive metric from a single illustrative seed; the multi-seed protocol and all statistical comparisons are based on MRE and GD. A second, within-dataset axis used leave-one-device-out on Aariz’s seven devices (train on six, test on the held-out device) with a leak-free, held-out-device-free validation set. This axis was run with the Vision Transformer, which showed the lowest absolute cross-domain error in the main matrix; device effects measured with the most transferable architecture are, therefore, a conservative estimate of what weaker architectures would show. A human-expert reference was obtained from senior-versus-junior inter-observer variability on Aariz.

### 2.5. Statistical Analysis

Architectures were grouped into two families: from-scratch heatmap models (HRNet and the two-stage cascade) and pretrained coordinate-regression models (Vision Transformer and ResNet-50). Each family, therefore, contributes four Generalization Drop estimates, one per architecture and transfer direction, each itself a mean over three seeds. We summarize these descriptively and compare the families by their observed separation rather than by a pooled significance test: the eight estimates derive from only two architectures and two directions, so treating them as independent observations would overstate the effective sample size. Where confidence intervals are reported for single quantities, they were obtained by bootstrap (10,000 resamples). The leave-one-device-out paired comparison used the Wilcoxon signed-rank test with a Shapiro–Wilk check of distributional assumptions. Effect sizes are reported alongside *p*-values. No a priori power calculation was performed: the number of devices is fixed by the Aariz dataset at seven, and the number of seeds was set by available compute rather than by a target power, so the leave-one-device-out comparison in particular should be read as descriptive.

The same three-seed protocol was applied to every experimental axis. In the ablation, a single coordinate-regression backbone (ResNet-50) was trained either from ImageNet-pretrained weights or from random initialization with all other settings held constant, isolating initialization as the only varying factor. ResNet-50 was chosen because it is the architecture in our set for which pretrained and randomly initialized versions are otherwise identical, making the swap a clean single-variable manipulation, and because it showed the smallest Generalization Drop in the main matrix—so it is the model whose transfer advantage most requires explanation.

## 3. Results

### 3.1. In-Domain Accuracy

Trained and tested within the same dataset (mean over three seeds), the four families reached MRE of 2.47–3.04 mm on Aariz and 4.12–5.55 mm on ISBI (Table 2, diagonal). On Aariz, the HRNet-style network was the most accurate in-domain (2.47 mm), followed closely by the Vision Transformer (2.67 mm) and the cascade (2.75 mm), with the ImageNet-pretrained ResNet-50 last (3.04 mm); the four families spanned only 0.57 mm in-domain, against a spread of 1.43 mm on the smaller single-device ISBI set.

**Table 2:** Cross-dataset mean radial error (mm), mean ± SD over three seeds. Each cell is labeled by training dataset then test dataset (e.g., Aariz→Aariz is trained on Aariz, tested on Aariz); A = Aariz, I = ISBI. Diagonal columns (A→A, I→I) are in-domain and off-diagonal columns (A→ISBI, I→Aariz) are cross-domain. The corresponding Generalization Drops are given in Table 3, and the same values are shown as a matrix in Supplementary Figure S1. SDR@2 mm from a single illustrative seed (secondary metric, not part of the multi-seed protocol) was 63.7% (HRNet), 59.8% (cascade), 46.1% (ViT), and 37.6% (ResNet-50) in-domain, falling to 5.8–17.4% cross-domain. Inter-observer reference was 0.53 mm.

Architecture	A→A (in)	A→ISBI	I→I (in)	I→Aariz
HRNet	2.47 ± 0.12	11.13 ± 1.54	4.12 ± 0.94	21.25 ± 1.83
Cascade	2.75 ± 0.31	12.01 ± 1.82	4.40 ± 0.48	22.15 ± 1.61
ViT	2.67 ± 0.09	8.67 ± 0.73	5.11 ± 0.29	20.15 ± 0.26
ResNet50	3.04 ± 0.12	7.73 ± 0.39	5.55 ± 0.14	23.07 ± 1.30

**Table 3:** Generalization Drop, GD = (MRE_cross − MRE_in)/MRE_in × 100, corresponding to the errors in Table 2; mean ± SD over three seeds. GD was computed separately within each seed and then averaged; because the average of a ratio is not the ratio of averages, these values differ from the ratio of the seed-averaged errors in Table 2 by up to about ten percentage points, with the largest divergence where seed variance is highest. GD A→I is the drop when training on Aariz and testing on ISBI; GD I→A is the reverse. The two from-scratch heatmap architectures occupy the four highest values and the two pretrained coordinate-regression architectures the four lowest, with no overlap between the families.

Architecture	GD A→I	GD I→A
HRNet	350 ± 44%	426 ± 70%
Cascade	338 ± 52%	408 ± 74%
ViT	225 ± 22%	295 ± 27%
ResNet50	155 ± 13%	316 ± 18%

### 3.2. Cross-Dataset Generalization (Axis 1)

**Figure 1:**
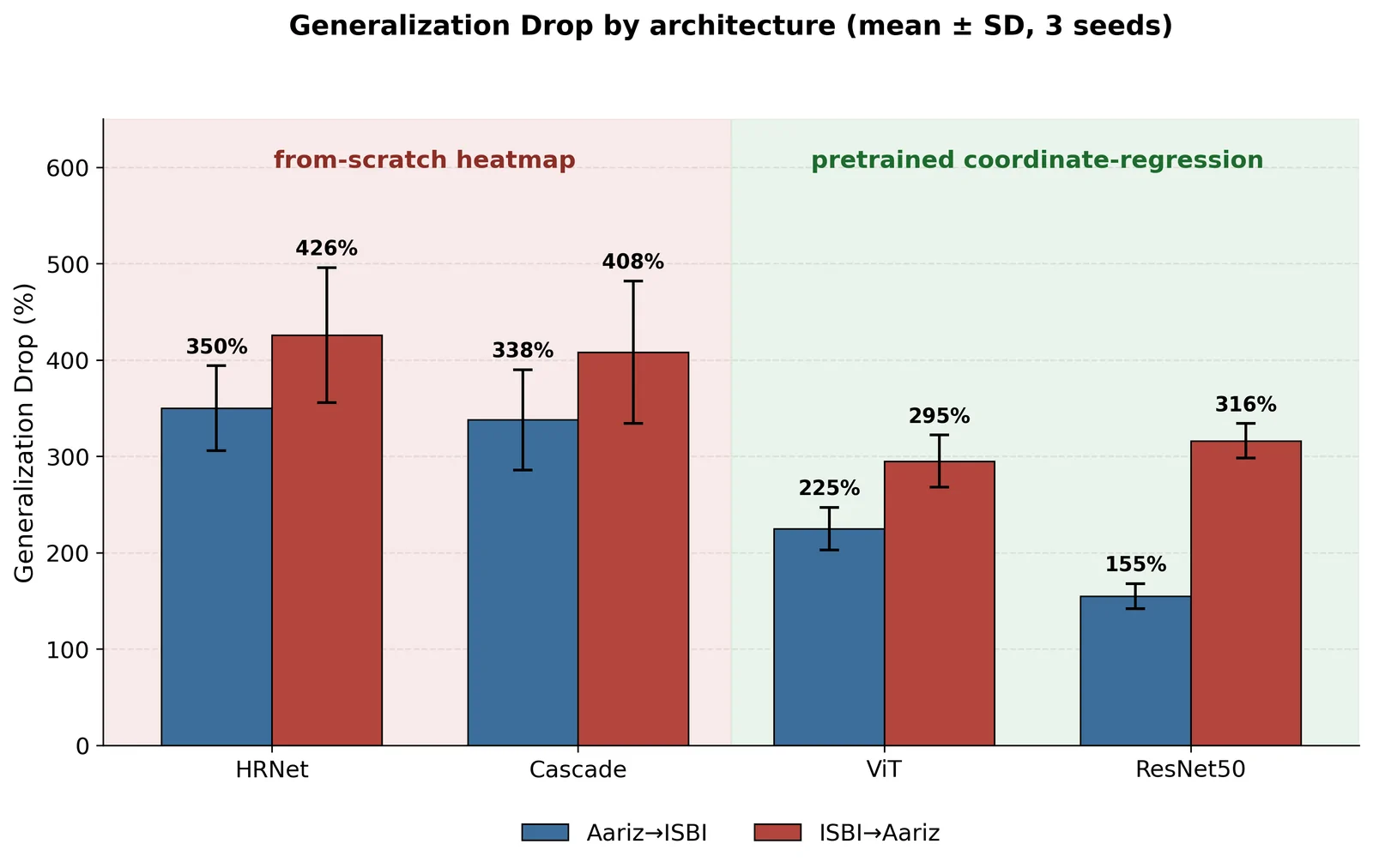
Generalization Drop (the percentage increase in MRE from in-domain to cross-domain testing) for each architecture in both transfer directions. Bars show the mean over three seeds and error bars the standard deviation across those seeds. The from-scratch heatmap models (HRNet, cascade) show the largest drops, including the HRNet, which was the most accurate model in-domain. The shaded background separates the two architecture families: from-scratch heatmap models (**left**) and pretrained coordinate-regression models (**right**).

**Figure 2:**
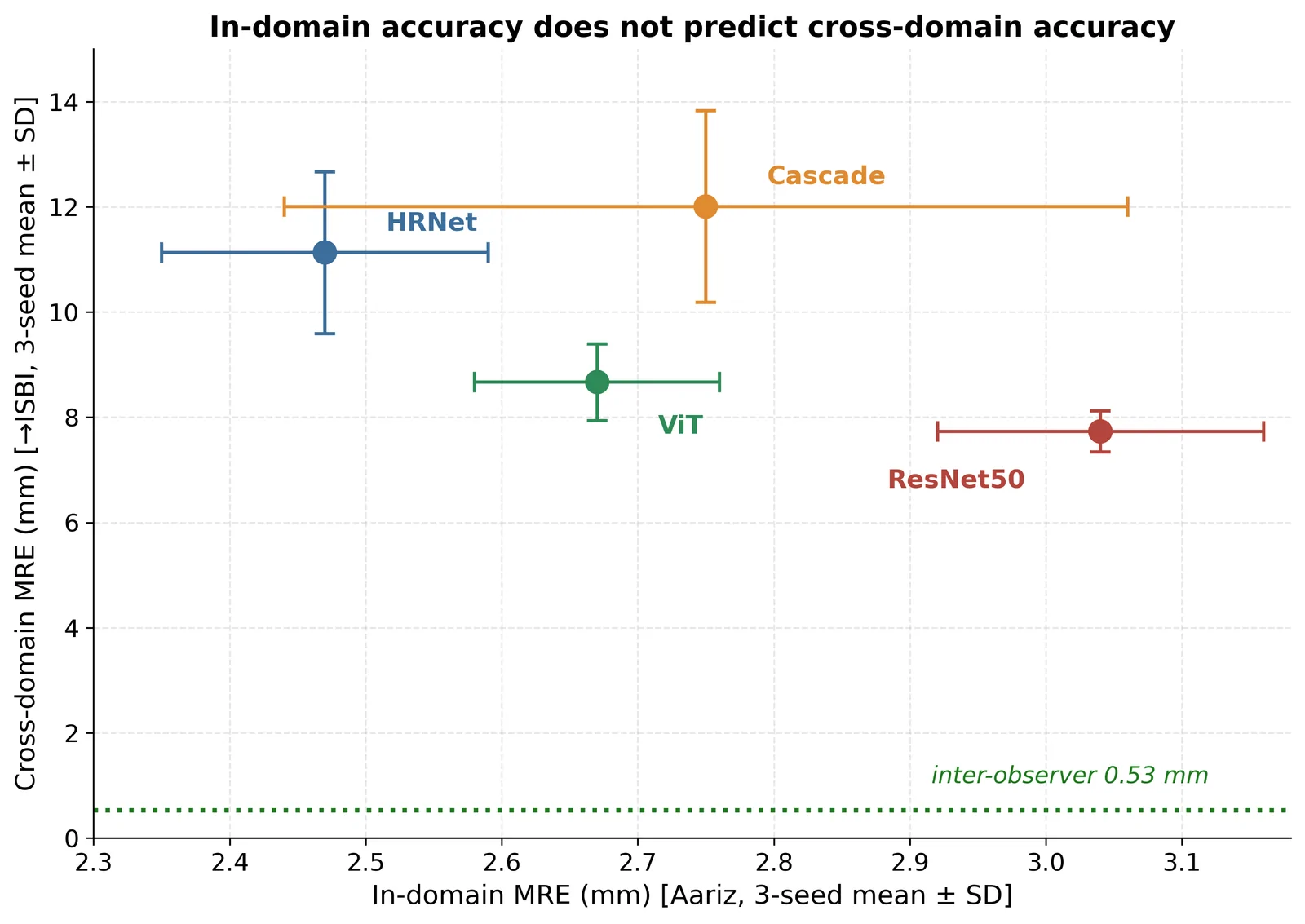
In-domain accuracy plotted against cross-domain accuracy for each architecture (MRE, mm; mean over three seeds, error bars show the standard deviation across seeds for both axes). The horizontal reference line marks the inter-observer band of 0.53 mm measured on Aariz. Architectures that rank best in-domain do not rank best out-of-domain, and every model lies far above the human reference when tested cross-domain.

**Figure 3:**
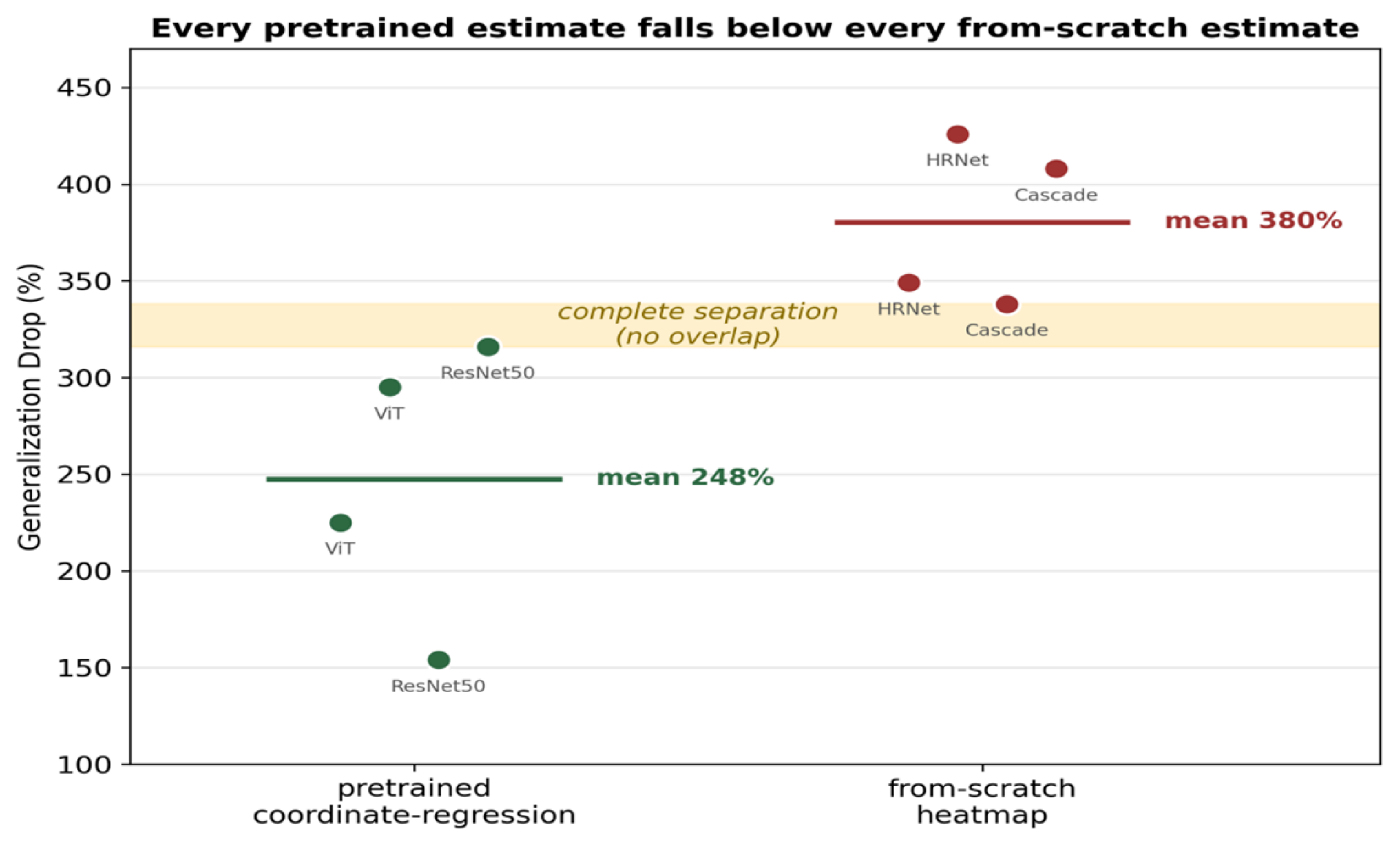
Generalization Drop by architecture family. Each family contributes four estimates (two architectures × two transfer directions), and each point is the mean over three seeds; horizontal lines mark the family means. The two families separate completely: the highest pretrained estimate (316%) lies below the lowest from-scratch estimate (338%), leaving the shaded gap with no overlapping values. Because the ordering is complete, the comparison does not depend on assumptions about the independence of individual runs.

Evaluated on the held-out dataset, error increased sharply in every case (Table 2, off-diagonal). Trained on Aariz and tested on ISBI, MRE rose from 2.47 to 11.13 mm (HRNet), 2.75 to 12.01 mm (cascade), 2.67 to 8.67 mm (ViT), and 3.04 to 7.73 mm (ResNet50). Trained on ISBI and tested on Aariz, the collapse was larger (20.15–23.07 mm). Figure 1 shows the Generalization Drop for each architecture and direction. Three patterns emerged. First, in-domain accuracy did not predict cross-domain accuracy (Figure 2): the in-domain and cross-domain orderings were almost exactly inverted. The most accurate model in-domain (HRNet, 2.47 mm) was the second worst on ISBI (11.13 mm), while the least accurate in-domain (ResNet-50, 3.04 mm) transferred best in this direction (7.73 mm); the from-scratch heatmap pair occupied the two worst cross-domain positions, including the architecture that was most accurate in-domain. Second, models trained on the multi-device Aariz set generalized far better than those trained on the single-device ISBI set, across all families; this is not a controlled contrast, however, since Aariz is also the larger set (1000 vs. 400 images) and differs in population and annotation protocol, so device diversity, training set size, and cohort cannot be separated here. Third, the two architecture families separated completely (Figure 3). Every one of the four pretrained estimates (one per architecture and direction; range 155–316%) fell below every one of the four from-scratch estimates (range 338–426%), with family means of 248% and 380%, respectively. Because this is a complete ordering, not an overlapping difference, it does not depend on any assumption about the independence of the underlying seeds; we, therefore, report it descriptively, since a pooled *p*-value would overstate the effective sample size. The pretrained models were also markedly more reproducible across seeds (mean per-cell SD ~20% vs. ~60% for the from-scratch models), indicating both better and more stable generalization.

### 3.3. Within-Dataset, Cross-Device Generalization (Axis 2)

**Figure 4:**
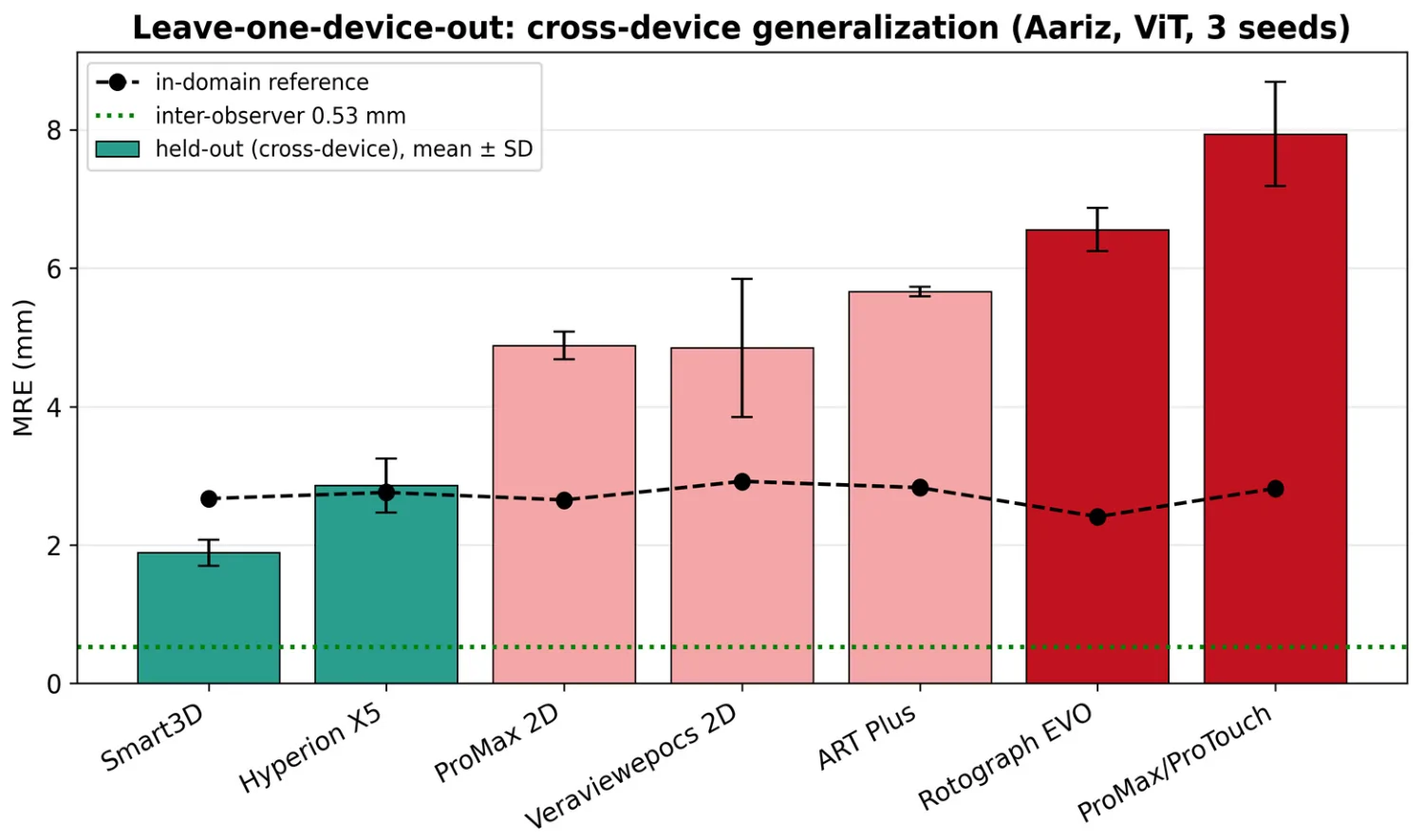
Leave-one-device-out results on Aariz using the Vision Transformer. For each of the seven imaging devices, the model was trained on the remaining six and tested on the held-out device; bars show the held-out MRE as the mean over three seeds and error bars the standard deviation across seeds. The in-domain reference and the 0.53 mm inter-observer band are marked for comparison. The effect is strongly device-dependent, ranging from better-than-in-domain performance (Smart3D) to more than a doubling of error (ProMax with ProTouch). Bar colour indicates the direction and size of the shift: green for devices that generalized better than or close to in-domain, pink for intermediate drops, and red for devices whose error more than doubled.

Leave-one-device-out on Aariz (seven devices, ViT, leak-free validation) showed domain shift even within a single dataset (Table 4, Figure 4). Held-out-device MRE averaged 4.95 mm against an in-domain reference of ~2.72 mm. This difference did not reach statistical significance (Wilcoxon signed-rank *p* = 0.078; Shapiro–Wilk *p* = 0.25), which we attribute to the small number of devices (*n* = 7) and the heterogeneity of the per-device effect, not to the absence of a shift; we, therefore, treat the leave-one-device-out result as indicative rather than confirmatory. The shift was device-specific, not uniform, and this pattern was reproducible across seeds. The seven devices fell into four bands (Table 4). One generalized better than in-domain in every seed (Smart3D, GD −29 ± 7%) and one was neutral on average but variable across seeds (Hyperion X5, GD 5 ± 21%). Three were intermediate, with drops of 66–101% (Veraviewepocs 2D, ProMax 2D, ART Plus), and two degraded consistently, more than doubling the in-domain error (Rotograph EVO 6.55 ± 0.30 mm, GD 172 ± 9%; ProMax with ProTouch 7.94 ± 0.75 mm, GD 181 ± 15%).

The multi-seed replication is informative in two ways. First, the extreme devices are highly reproducible: the best-transferring device (Smart3D) and the two worst (Rotograph EVO, ProMax with ProTouch) show narrow seed-to-seed variation (GD SD ≤ 15 percentage points), so these are genuine device effects rather than sampling noise. Second, the near-neutral device (Hyperion X5) is precisely the one with the widest spread (GD 5 ± 21%, ranging from −8% to +29% across seeds), which illustrates why single-run device analyses can mislead: a single seed would have supported either “no shift” or “moderate shift” for this device. Held-out sets for the two smallest devices were modest (ProMax 2D *n* = 41, Smart3D *n* = 59), but their seed-to-seed variability was among the lowest in the panel (GD SD 11 and 7 percentage points, respectively), indicating that their estimates are not dominated by small-sample noise; the overall pattern holds across all seven devices spanning *n* = 41–366.

### 3.4. Human-Expert Reference (Inter-Observer Variability)

Senior-versus-junior orthodontist disagreement on Aariz (1000 images, 19 landmarks, 19,000 paired measurements) averaged 0.53 mm (median 0.13 mm; per-landmark means ranged 0.06–1.34 mm; 93.9% of measurements within 2 mm). The right-skewed distribution (mean > median) reflects a few harder landmarks dominating the mean. The mean model error did not approach the mean inter-observer difference even in-domain (best ~2.5 mm, ~5× the 0.53 mm reference), and cross-domain mean errors were 15–44× that reference. The two quantities are not strictly commensurable: the inter-observer figure is the spread between two human tracings of the same image, whereas the model is scored against a fixed ground truth, so the model is held to the stricter standard. The least reproducible landmarks were Porion (1.34 mm) and Gonion (1.32 mm), both bilateral structures whose projection was ambiguous on a lateral radiograph; the most reproducible was Sella (0.06 mm). Two caveats are attached to this reference. First, the inter-observer band is derived from Aariz alone, because ISBI’s public export contains a single tracing; comparing ISBI cross-domain error against an Aariz-derived human band is, therefore, not strictly like-for-like, and the “15–44×” figure should be read as an order-of-magnitude reference rather than a matched comparison. Second, imperfect observer agreement is a general feature of two-dimensional radiographic interpretation in orthodontics and has been documented for other diagnostic tasks on the same imaging modality [17]; this underlines the difficulty of defining a flawless ground truth for automated landmark detection.

### 3.5. Mitigation: Balanced Joint Training (Axis 3)

**Figure 5:**
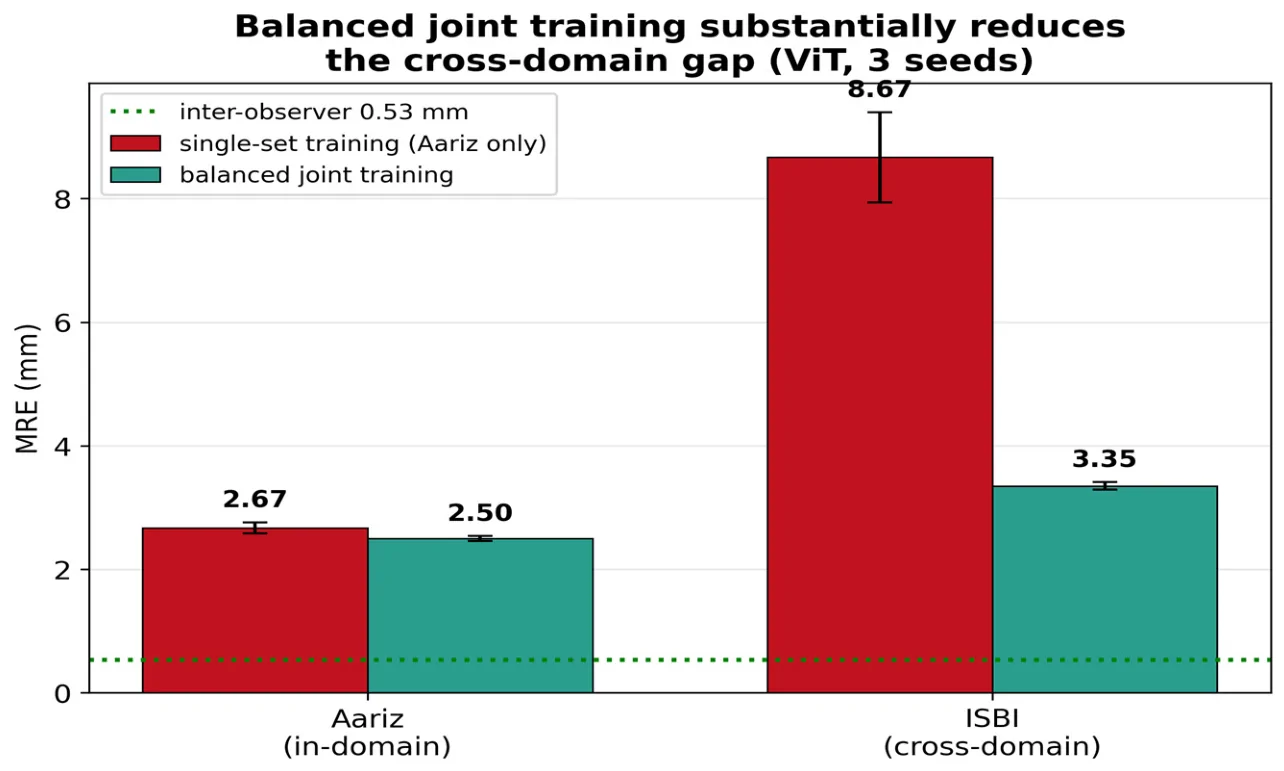
Effect of balanced joint training, illustrated with the Vision Transformer. Bars compare MRE on each dataset after single-set training (trained on Aariz only) against balanced joint training on both datasets; values are means over three seeds and error bars show the standard deviation across seeds. Joint training brings cross-domain error close to in-domain levels without degrading in-domain accuracy.

Training on a balanced mixture of both datasets substantially reduced the cross-domain gap for every architecture (Table 5, Figure 5; mean ± SD over three seeds per condition). In every case, both domains landed near in-domain accuracy (≈2.1–3.5 mm) without degrading in-domain performance; HRNet, for example, was slightly better on Aariz under joint training than under single-set training (2.21 ± 0.17 mm vs. 2.47 ± 0.12 mm). The from-scratch heatmap models, most fragile under single-set training, became the strongest under joint training (2.53 ± 0.13 mm for HRNet and 2.52 ± 0.05 mm for the cascade on ISBI), indicating that the training distribution is a major driver of cross-domain error, although architecture effects do not vanish entirely under joint training.

### 3.6. Pretrained-Versus-Scratch Ablation

To test the proposed mechanism directly, we repeated the cross-dataset experiment with a single architecture (coordinate-regression ResNet-50) under two initializations—ImageNet-pretrained and trained from scratch—holding every other setting constant, over three seeds. The pretrained arm uses the same configuration as the ResNet-50 reported in Table 2, so its agreement with that row (in-domain 3.04 mm and cross-domain 7.73 ± 0.39 mm in both) serves as an internal consistency check rather than as an independent result; the scratch arm is the new comparison.

Pretraining improved in-domain accuracy on Aariz (3.04 mm vs. 3.91 mm from scratch) and produced a large advantage in the Aariz-to-ISBI direction: cross-domain error was 7.73 ± 0.39 mm with pretraining versus 16.36 ± 0.29 mm from scratch, a 2.1-fold reduction, with Generalization Drop 155 ± 13% versus 318 ± 9%. In the opposite direction (ISBI to Aariz), the two initializations performed similarly in absolute terms (23.07 ± 1.30 mm pretrained vs. 20.62 ± 0.22 mm from scratch), and the Generalization Drop was in fact higher for the pretrained model (316 ± 19% vs. 259 ± 4%).

This asymmetry requires care in interpretation. Because Generalization Drop is a ratio normalized by in-domain error, a model that is better in-domain is penalized in the ratio when its absolute cross-domain error is comparable; the pretrained model’s stronger in-domain performance, therefore, inflates its drop in the ISBI-to-Aariz direction even though absolute errors are similar. Averaged over both directions, the pretrained model showed a lower mean drop (235 ± 90%) than the scratch model (288 ± 33%), but the spread is wide, and the intervals overlap.

We, therefore, report this ablation as partial rather than conclusive support for the pretraining mechanism. It establishes that ImageNet initialization confers a substantial and reproducible transfer advantage when training on the larger, multi-device dataset and testing on the single-device one, but not that pretraining improves transfer symmetrically in all directions.

### 3.7. Landmark-Level Breakdown

Aggregate MRE conceals substantial variation between anatomical points, and clinical tolerance is landmark-specific. We, therefore, repeated the cross-dataset experiment for two architectures chosen to bracket the effect: the from-scratch HRNet-style network, which was the most accurate in-domain yet showed the largest Generalization Drop (388% averaged over both directions), and the Vision Transformer, which had the lowest absolute cross-domain error (14.41 mm). The error was recorded separately for each of the 19 shared landmarks over three seeds (full table in Supplementary Table S1). These models were retrained under the identical configuration used for the main matrix, and their cross-domain aggregates reproduce it exactly (11.13 mm for the heatmap network and 8.67 mm for the ViT). The exact agreement confirms that the pipeline is deterministic; the breakdown, therefore, resolves the same predictions to landmark level rather than providing independent corroboration. In-domain aggregates ran slightly higher than in Table 2 (2.58 vs. 2.47 mm and 2.94 vs. 2.67 mm), reflecting seed variation and the fact that the mean of per-landmark means weights landmarks equally; since the clinical comparison below concerns cross-domain error, this does not affect the conclusions drawn.

For the clinically load-bearing points, the answer is unambiguous. A-point, B-point, Nasion, and Menton all exceeded the 2 mm clinical threshold under domain shift, in both architectures and without exception: for the heatmap network, they reached 12.06, 10.57, 7.67, and 5.96 mm, respectively, and for the ViT, they reached 11.92, 5.96, 11.64, and 4.96 mm. Several of these points were comfortably acceptable in-domain—Menton, for instance, was measured at 0.73 ± 0.02 mm by the heatmap network, well inside the 2 mm threshold and among the most precise in-domain values we recorded—yet none remained within tolerance out-of-domain. Cross-domain error is, therefore, not confined to intrinsically difficult landmarks; it reaches the points on which skeletal classification depends.

The breakdown also shows that the two architectures fail in different ways. The heatmap network is highly uneven: in-domain, it spans 0.72 mm (Gnathion) to 5.05 mm (Gonion), and cross-domain, it spans 3.89 mm to 29.21 mm, with Gonion, Porion, Articulare, and Sella deteriorating most. The ViT is far more uniform, spanning 2.13–3.90 mm in-domain and 4.77–12.61 mm cross-domain; at Gonion, the difference between the two is more than threefold (8.04 mm versus 29.21 mm). Notably, the three landmarks the heatmap network localized best in-domain—Gnathion (0.72 mm), Menton (0.73 mm), and Pogonion (1.10 mm)—were also among those with the largest relative degradation (Generalization Drop 626%, 716%, and 661%). Across all 19 landmarks, the rank correlation between in-domain error and Generalization Drop was not statistically significant (Spearman ρ = −0.19, *p* = 0.42), so we do not claim a general inverse relationship; we note only that the architecture-level pattern reported above—sub-millimeter in-domain precision accompanying the steepest collapse—is visible at the level of individual landmarks as well.

Finally, the landmarks that degraded most under domain shift overlap with those that human observers agree on least: Porion and Gonion, which showed the highest inter-observer variability in Section 3.4 (1.34 mm and 1.32 mm), were also among the worst cross-domain points for both architectures. Anatomical ambiguity and sensitivity to acquisition shift, therefore, appear to affect the same structures.

## 4. Discussion

***Principal findings.*** Across four architecture families and two datasets, in-domain superiority did not transfer: the single most accurate model in-domain (the from-scratch HRNet, 2.47 mm on Aariz) was among the worst across datasets, and the from-scratch heatmap family as a whole showed the largest Generalization Drops, while the pretrained coordinate-regression models transferred best despite the least accurate of them ranking last in-domain. The separation between families was complete: all four pretrained estimates fell below all four from-scratch estimates (family means 248% vs. 380%). Generalization degraded in every direction and at every scale tested—between datasets and even between devices within a single dataset—and balanced multi-source training recovered most of the loss for every architecture.

The practical implication of this pattern is worth stating plainly. If in-domain leaderboards do not predict cross-domain ranking, then the standard practice of selecting an architecture on the basis of single-dataset performance is unreliable for any application that will encounter images from new devices or centers—which is to say, essentially all clinical deployment. A model chosen because it tops the ISBI leaderboard may be among the worst choices once it meets images from a different scanner. This inversion, not any single error value, is the central message of our results.

***Comparison with prior work.*** Single-dataset reports approach ~1.2–1.5 mm on ISBI [4]; our in-domain figures are higher because every architecture used one fixed training budget for fair comparison rather than per-model tuning, and because we report on a strict shared-landmark subset in millimeters. The out-of-domain drop is consistent with the broader literature: the CL-Detection 2023 organizers questioned whether single-center, single-vendor benchmarks generalize and built a multi-center resource for that reason [4], and transfer learning baselines evaluated on a diverse cephalometric dataset alongside the ISBI benchmark report substantial differences in accuracy between architectures and across image subcategories [18]. Where external validation exists, reported drops are typically milder than ours: a multi-center three-dimensional model retained approximately 2 mm accuracy across Finnish and Thai cohorts [19]. Our larger drops reflect a deliberately harsher, fully independent, untuned test rather than a contradiction.

***Architecture and generalization.*** The Vision Transformer, achieving the lowest average cross-domain error (14.41 mm across the two directions), despite ranking second on Aariz and third on ISBI in-domain, aligns with evidence that ViTs are more robust than CNNs under distribution shift [20]. Whether that robustness stems from self-attention itself is contested: convolutional networks with suitable design choices have been shown to match or exceed transformer robustness [21], which shifts the explanation toward training regime and architectural detail rather than attention per se. Our own results point the same way, since both of our best out-of-domain models were pretrained coordinate-regression networks—one transformer and one convolutional—and the pretrained-versus-scratch ablation isolated initialization, not architecture, as the operative factor. Across three seeds, the two families separated completely, with no overlap between their Generalization Drop estimates, and the pretrained models were additionally threefold more reproducible across seeds, strengthening this beyond a single-run observation. The mitigation result echoes the domain generalization and domain adaptation literature, which identifies multi-source training as a first-line strategy [8,9], and complements the single-source line of work that instead expands training diversity through aggressive augmentation when only one domain is available [22].

A plausible mechanism ties these observations together. Large-scale pretraining exposes a network to enormous natural image variability before it ever sees a cephalogram, yielding low-level and mid-level features that are comparatively invariant to the intensity and texture idiosyncrasies of a particular scanner. A from-scratch heatmap network, by contrast, learns all of its features from a few hundred images acquired on one device, and can achieve very sharp in-domain localization precisely by exploiting device-specific regularities that do not generalize. On this view, the strong in-domain performance of the scratch models and their poor transfer are two faces of the same phenomenon: features finely tuned to one acquisition distribution. The coordinate-regression formulation of our pretrained models may further help, because directly regressing coordinates couples less tightly to the absolute pixel-intensity patterns that shift most across devices than dense heatmap matching does. To move this from interpretation toward evidence, we performed a controlled pretrained-versus-scratch ablation on an identical architecture (Section 3.6), holding the network fixed and varying only ImageNet initialization. It shows that pretraining confers a large and reproducible advantage when transferring from the multi-device dataset to the single-device one (cross-domain error 7.73 ± 0.39 mm vs. 16.36 ± 0.29 mm from scratch), while in the reverse direction the two initializations perform comparably; the mechanism is, therefore, supported in one transfer direction rather than uniformly, a claim the ablation tests directly instead of assuming.

***Clinical interpretation.*** Inter-observer variability here was 0.53 mm, and a 2 mm radial error is a common clinical acceptability threshold. In-domain, the best model sat at ~2.5 mm (SDR@2 mm 63.7% on the illustrative seed); cross-domain, SDR@2 mm fell to 6–17%, i.e., fewer than one in five landmarks within tolerance—unusable for unsupervised deployment on a new device or center. The device-specific leave-one-device-out result implies that a model validated on one clinic’s scanner may fail on another’s, and that the devices represented in training should be chosen deliberately. The mitigation result offers a concrete, low-cost remedy: pooling balanced data from the target imaging sources restored ≈ 2.1–3.5 mm across both domains.

These findings carry a specific message for anyone building or procuring such systems. Vendor-reported accuracy, if obtained on a single internal dataset, should be treated as an upper bound that may not hold on local images; acceptance testing on the receiving center’s own devices is essential before clinical reliance. Where a system must serve multiple sites, our mitigation result suggests that assembling even a modest, balanced sample from each target device and retraining is a more dependable route to robustness than selecting a nominally superior architecture trained elsewhere. The broader literature on safe and responsible medical AI stresses caution before clinical reliance on models validated in narrow settings [23,24]. Our results provide a concrete quantitative illustration of why in-domain metrics alone are insufficient evidence of fitness for deployment.

***Limitations.*** All four experimental axes—the cross-dataset matrix, the leave-one-device-out analysis, the joint-training mitigation, and the pretrained-versus-scratch ablation—were repeated over three seeds and are reported as mean ± SD. Three seeds nevertheless remain a modest number, and broader replication across more seeds, datasets, and centers would further tighten the estimates; the per-device estimates in particular carry visible seed-to-seed variability for some devices.

A second constraint is the fixed training recipe. Holding the optimizer, schedule, input resolution, and epoch budget constant across architectures equalizes experimenter effort, but it does not isolate architecture as a causal factor, and it means the from-scratch heatmap models were not tuned to their individual optima. Their large generalization drop should, therefore, be read as the behavior of these architectures under a common budget, not as a demonstrated architectural ceiling; under-optimization and intrinsic architectural limits cannot be fully separated in this design. The pretrained-versus-scratch ablation partially addresses this by holding the architecture fixed and varying only initialization, but a full per-architecture tuning study remains outside the scope of this work.

A third constraint is analysis coverage: two analyses were run with a single architecture rather than all four—the leave-one-device-out axis used the Vision Transformer, and the landmark-level breakdown in Section 3.7 used the two extreme architectures on a single training source (Aariz). The device-level behavior of the remaining architectures is, therefore, not characterized, although running the device axis with the most transferable model makes those estimates conservative. The landmark-level analysis likewise establishes that the clinically load-bearing points exceed the 2 mm threshold under domain shift for the two architectures examined but does not describe the full landmark-by-architecture surface; extending it to the cascade and the pretrained ResNet-50, and to both transfer directions, would complete that picture.

ISBI ground truth is senior-only in the public export, whereas Aariz uses the senior + junior mean; this difference in ground truth definition may contribute to the apparent cross-dataset gap. To bound its magnitude, we note that the reported inter-observer variability on these landmarks is ~0.53 mm, so the senior-versus-mean discrepancy is on the order of a few tenths of a millimeter—more than an order of magnitude smaller than the cross-dataset shifts of 5–18 mm we observe. The ground truth asymmetry, therefore, cannot account for the bulk of the generalization gap, though we retain it as a limitation and note that harmonized annotation would tighten the estimates. Relatedly, the two datasets differ not only in acquisition equipment but also in the patient populations they were drawn from, so the cross-dataset gap reported here reflects the combined effect of device, annotation protocol, and population morphology rather than acquisition shift alone; disentangling these components would require datasets matched on one factor while varying another, which the available public resources do not currently permit.

Several further limitations should be noted. Architecture is confounded with pretraining, output representation, and input resolution: the two families that transferred best were both pretrained, both coordinate-regression, and both trained at 224 × 224, so these attributes cannot be disentangled from one another in the main matrix. The ablation in Section 3.6 isolates initialization alone and does not resolve the remaining confounds. No inferential test was applied at the landmark level; the breakdown in Section 3.7 is descriptive, and the rank correlation reported there was not significant. Downstream cephalometric measurements such as SNA, SNB, and ANB were not computed, so the propagation of landmark error into the angular quantities used clinically remains to be quantified. The shared-landmark mapping was confirmed by a single orthodontist who is an author of this work; independent confirmation by two blinded orthodontists with a reported agreement statistic would be preferable, particularly for the soft tissue points.

Finally, the cross-dataset axis rests on two datasets. Two domains are sufficient to demonstrate that in-domain ranking fails to predict cross-domain ranking, but not to characterize how the gap scales with the number or diversity of source domains; incorporating further public datasets as they become available is a natural extension, and the within-Aariz leave-one-device-out analysis, which spans seven devices, partly compensates by providing a second and finer-grained shift axis.

## 5. Conclusions

The architecture that performs best on a single cephalometric dataset is not the one that generalizes best to another: in-domain accuracy and cross-domain robustness diverged, with the most accurate in-domain model (a from-scratch heatmap CNN) dropping most across datasets, and the pretrained coordinate-regression models transferring best—the Vision Transformer with the lowest absolute cross-domain error and the ResNet-50 with the smallest Generalization Drop. Domain shift was pervasive—between datasets and between devices within a single dataset—and left all models far above the 0.53 mm inter-observer band when tested out-of-domain. Resolved to individual landmarks, the effect reached the points that carry diagnostic weight: A-point, B-point, Nasion, and Menton all exceeded the 2 mm clinical threshold out-of-domain, including points localized to sub-millimeter accuracy on the training dataset. A controlled ablation showed that ImageNet pretraining accounts for part of this advantage, though not symmetrically in both transfer directions. Crucially, a simple balanced multi-source training scheme restored near-in-domain accuracy for every architecture, indicating that the training distribution is a primary driver of cross-domain error, even though architecture effects persist. Cephalometric AI should, therefore, be evaluated and trained across the imaging sources it will encounter clinically, not on a single benchmark. Three practical consequences follow. The field would benefit from standardized multi-device benchmarks rather than a single canonical test split; device-specific performance should be reported alongside aggregate accuracy, since the aggregate concealed a range from better-than-in-domain to more than doubled error across the seven scanners examined here; and before clinical reliance, a system should be validated on the receiving center’s own images and, where accuracy is insufficient, fine-tuned on a modest balanced sample from the target devices.

## Figures and Tables

**Table 1:** The 19 shared landmarks and their symbols in each dataset. Mappings for landmarks 13, 14, and 16 were confirmed by an orthodontist.

#	Landmark (Anatomical Name)	ISBI	Aariz
1	Sella	S	S
2	Nasion	N	N
3	Orbitale	Or	Or
4	Porion	Po	Po
5	Subspinale (A-point)	A	A
6	Supramentale (B-point)	B	B
7	Pogonion	Pog	Pog
8	Menton	Me	Me
9	Gnathion	Gn	Gn
10	Gonion	Go	Go
11	Lower incisor tip	LIT	LIT
12	Upper incisor tip	UIT	UIT
13	Upper lip (Labrale superius)	Ls	Ls
14	Lower lip (Labrale inferius)	Li	Li
15	Subnasale	Sn	Sn
16	Soft tissue Pogonion	Pog’	Pog’
17	Posterior nasal spine	PNS	PNS
18	Anterior nasal spine	ANS	ANS
19	Articulare	Ar	Ar

**Table 4:** Leave-one-device-out on Aariz (Vision Transformer). MRE in mm; mean ± SD over three seeds per configuration. GD = Generalization Drop. * SDR was not recomputed in the multi-seed replication and is, therefore, not reported here.

Held-Out Device	*n*	In-Domain	Held Out	SDR@2 mm *	GD
Smart3D	59	2.67 ± 0.02	1.89 ± 0.18	—	−29 ± 7%
Hyperion X5	143	2.76 ± 0.19	2.86 ± 0.39	—	5 ± 21%
ART Plus	366	2.83 ± 0.27	5.66 ± 0.07	—	101 ± 21%
Veraviewepocs 2D	177	2.92 ± 0.12	4.85 ± 1.00	—	66 ± 28%
ProMax 2D	41	2.65 ± 0.15	4.88 ± 0.20	—	85 ± 11%
ProMax with ProTouch	135	2.82 ± 0.16	7.94 ± 0.75	—	181 ± 15%
Rotograph EVO	79	2.41 ± 0.15	6.55 ± 0.30	—	172 ± 9%

**Table 5:** Balanced joint training vs. single-set training (MRE, mm; mean ± SD over three seeds for both conditions). Single-set columns are reproduced from Table 2 (train on Aariz; in-domain and cross-domain to ISBI). Joint training substantially reduces the cross-domain gap for every architecture while leaving in-domain accuracy essentially unchanged.

Architecture	Single-Set Aariz	Single-Set ISBI (Cross)	Joint Aariz	Joint ISBI
HRNet	2.47 ± 0.12	11.13 ± 1.54	2.21 ± 0.17	2.53 ± 0.13
Cascade	2.75 ± 0.31	12.01 ± 1.82	2.10 ± 0.11	2.52 ± 0.05
ViT	2.67 ± 0.09	8.67 ± 0.73	2.50 ± 0.04	3.35 ± 0.06
ResNet50	3.04 ± 0.12	7.73 ± 0.39	2.75 ± 0.10	3.54 ± 0.17

## Data Availability

No new data were generated in this study. The datasets analyzed are publicly available from their original sources: the ISBI 2015 Cephalometric X-ray Landmark Detection Challenge dataset (CC BY-SA 4.0) and the Aariz dataset (Figshare, DOI 10.6084/m9.figshare.27986417, CC-BY). No images were redistributed. The complete analysis code that produces every result reported here—including the multi-seed replications, the pretrained-versus-scratch ablation, and the landmark-level breakdown—is provided as Supplementary Material, together with Supplementary Table S1.

## Supplementary Materials

The following supporting information can be downloaded at: https://www.mdpi.com/article/10.3390/diagnostics16172726/s1, Figure S1: Cross-dataset error matrix; Table S1: Landmark-level error under domain shift; analysis code (Python scripts reproducing every reported result).
