# Supplementary material for: Cross-Domain Generalization of Deep Learning Architectures for Cephalometric Landmark Detection: A Dual-Dataset and Multi-Device Benchmark
Source: Diagnostics (Basel). 2026 Aug 26;16(17):2726. doi: 10.3390/diagnostics16172726 (PMC13564661; doi:10.3390/diagnostics16172726)
Supplement: Supplementary file 1 [file diagnostics-16-02726-s001.zip › supplementary_material.pdf]

Supplementary Material

Manuscript: Cross-Domain Generalization of Deep Learning Architectures for Cephalometric Landmark Detection: A Dual-Dataset and Multi-Device Benchmark

Figure S1. Cross-dataset error matrix

Cross-dataset mean radial error (MRE, mm) for each architecture, shown as a 2×2 matrix of training dataset (rows) against test dataset (columns). Diagonal cells are in-domain performance and off-diagonal cells are cross-domain performance; each value is the mean over three seeds. This figure presents the same values as Table 2 of the main text in visual form and is provided here for readers who prefer the matrix view.

[Figure S1 image: FigureS1_cross_matrix.png, supplied separately]

Table S1. Landmark-level error under domain shift

Per-landmark mean radial error (mm) for the two extreme architectures, trained on Aariz and evaluated in-domain (Aariz test split) and cross-domain (ISBI). Values are mean ± SD over three random seeds. GD = Generalization Drop, the percentage increase from in-domain to cross-domain error; as in Table 3 of the main text, GD was computed separately within each seed and then averaged, so individual values may differ by a few percentage points from the ratio of the seed-averaged errors shown. Landmark names and ordering follow Table 1 of the main text. Landmarks marked with an asterisk are those highlighted during review as clinically load-bearing. Aggregate values reproduce the cross-dataset matrix in Table 2 of the main text (cross-domain 11.13 mm and 8.67 mm respectively).

Landmark	Symbol	HRNet in	HRNet cross	HRNet GD	ViT in	ViT cross	ViT GD
Sella	S	4.08 ± 0.34	16.91 ± 5.25	314%	3.40 ± 0.21	11.22 ± 0.41	230%
**Nasion ***	N	2.99 ± 0.29	7.67 ± 2.01	157%	3.13 ± 0.13	11.64 ± 1.19	272%
Orbitale	Or	3.31 ± 0.34	9.42 ± 0.82	185%	3.41 ± 0.17	9.64 ± 1.09	182%
Porion	Po	4.28 ± 0.33	21.16 ± 1.98	394%	3.60 ± 0.31	12.01 ± 0.84	234%
**Subspinale (A-point) ***	A	2.70 ± 0.27	12.06 ± 0.94	346%	2.61 ± 0.11	11.92 ± 1.42	357%
**Supramentale (B-point) ***	B	1.95 ± 0.15	10.57 ± 3.12	442%	2.41 ± 0.10	5.96 ± 0.43	148%
Pogonion	Pog	1.10 ± 0.09	8.36 ± 5.14	661%	2.30 ± 0.11	5.12 ± 0.60	123%
**Menton ***	Me	0.73 ± 0.02	5.96 ± 3.25	716%	2.13 ± 0.12	4.96 ± 0.64	133%
Gnathion	Gn	0.72 ± 0.03	5.23 ± 3.79	626%	2.20 ± 0.08	4.77 ± 0.47	117%

Landmark	Symbol	HRNet in	HRNet cross	HRNet GD	ViT in	ViT cross	ViT GD
Gonion	Go	5.05 ± 0.46	29.21 ± 0.96	479%	3.90 ± 0.23	8.04 ± 1.24	106%
Lower incisor tip	LIT	2.61 ± 0.21	10.67 ± 2.20	309%	2.80 ± 0.20	7.75 ± 1.62	176%
Upper incisor tip	UIT	1.84 ± 0.48	4.75 ± 2.24	158%	2.75 ± 0.06	6.69 ± 0.74	144%
Upper lip (Labrale superius)	Ls	1.59 ± 0.38	3.89 ± 1.04	145%	3.00 ± 0.14	8.34 ± 1.15	178%
Lower lip (Labrale inferius)	Li	1.36 ± 0.12	4.36 ± 1.76	220%	2.94 ± 0.19	10.12 ± 1.72	244%
Subnasale	Sn	2.54 ± 0.26	5.27 ± 0.34	108%	2.89 ± 0.07	9.30 ± 0.83	222%
Soft-tissue Pogonion	Pog'	1.71 ± 0.29	14.41 ± 4.77	743%	2.81 ± 0.06	12.61 ± 2.19	348%
Posterior nasal spine	PNS	3.53 ± 0.14	15.69 ± 1.72	345%	3.23 ± 0.09	7.96 ± 0.98	147%
Anterior nasal spine	ANS	3.05 ± 0.26	6.72 ± 0.94	120%	3.04 ± 0.10	7.51 ± 0.88	147%
Articulare	Ar	3.93 ± 0.22	19.25 ± 0.93	390%	3.33 ± 0.20	9.16 ± 0.32	175%
**All 19 (mean)**	**—**	**2.58**	**11.13**	**331%**	**2.94**	**8.67**	**195%**

Notes. All four clinically load-bearing landmarks exceed the 2 mm clinical threshold under domain shift in both architectures, including landmarks that were within or near that threshold in-domain (for example Menton at 0.73 ± 0.02 mm for the heatmap network). The heatmap network shows markedly greater heterogeneity across landmarks than the Vision Transformer, both in-domain (0.72–5.05 mm versus 2.13–3.90 mm) and cross-domain (3.89–29.21 mm versus 4.77–12.61 mm). Porion and Gonion, which showed the highest inter-observer variability in Section 3.4 of the main text, are also among the landmarks that degrade most under domain shift.
